# Low-toxicity FePt nanoparticles for the targeted and enhanced diagnosis of breast tumors using few centimeters deep whole-body photoacoustic imaging

**DOI:** 10.1016/j.pacs.2020.100179

**Published:** 2020-04-11

**Authors:** Yubin Liu, Pei-Chun Wu, Sen Guo, Pi-Tai Chou, Chuxia Deng, Shang-Wei Chou, Zhen Yuan, Tzu-Ming Liu

**Affiliations:** a Cancer Center, Faculty of Health Sciences, University of Macau, Macau SAR, China; b Institute of Translational Medicine, Faculty of Health Sciences, University of Macau, Macau SAR, China; c Department of Chemistry, National Taiwan University, Taipei, 10617, Taiwan

**Keywords:** Whole-body photoacoustic imaging, Breast cancer, Imaging depth, FePt nanoparticles

## Abstract

A considerable amount of early breast tumors grown at a depth over 2 cm in breast tissues. With high near-infrared absorption of iron-platinum (FePt) nanoparticles, we achieved few centimeters deep photoacoustic (PA) imaging for the diagnosis of breast tumors. The imaging depth can extend over 5 cm in chicken breast tissues at the low laser energy density of 20 mJ/cm^2^ (≤ ANSI safety limit). After anti-VEGFR conjugation and the tail-vein injection, we validated their targeting on tumor sites by the confocal microscopy and PA imaging. Using a home-made whole-body *in vivo* PA imaging, we found that the nanoparticles were rapidly cleared away from the site of the tumor and majorly metabolized through the liver. These results validated the clinical potential of the FePt nanoparticles in the low-toxicity PA theragnosis of early breast cancer and showed the capacity of our whole-body PA imaging technique on monitoring the dynamic biodistribution of nanoparticles in the living body.

## 1 Introduction

Breast cancer is the second most common type of cancer after lung cancer worldwide, and one in four women in the United States will develop breast cancer during their lifetime. As the predominant conventional approach for breast cancer detection, annual mammography screening had been introduced to public. While x-ray mammography is the current clinical tool for screening and diagnosis of breast cancer, it has numerous limitations such as the inability of imaging dense breast and the use of ionization radiation. To date, among various techniques being developed to break the basic limitations of x-ray imaging, diffuse optical tomography (DOT) and photoacoustic imaging (PAI) are particularly interesting and promising. DOT can provide both tissue structural and functional information at a depth of several centimeters. However, the 5−10 mm spatial resolution of DOT is hard to identify few millimeters sized lumps in breast tissues, which is critical for the early diagnosis of breast tumor [1]. By contrast, PAI used acoustic detection to overcome the problem of light scattering and offer sub-millimeter resolution of anatomical, functional and molecular imaging at few centimeters imaging depth [[2], [3], [4]]. Combined with the rich endogenous and exogenous optical contrasts of absorption, PAI has been successfully applied to the early detection of cancer. It is reported that PAT can achieve 5 cm *in vitro* imaging depth in chicken breast tissues theoretically [5]. The imaging depth reduced to around 2 cm for whole-body imaging of mice *in vivo* [6,7]. Interestingly, a considerable amount of early breast tumors usually grown at a depth over 2 cm in breast tissues [[8], [9], [10]], which can be reached and resolved by PAI. Therefore, PAI is a very suitable imaging modality for the early detection of breast cancer *in vivo*. After finding suspected lumps in breast tissues, to increase the sensitivity of detection, the specificity of diagnosis, and the depth of exploration, exogenous contrast agents for near-infrared PAI are necessary. By far, many contrast agents have been developed for boosting the performance of PAI [[11], [12], [13], [14], [15]]. The goal is to obtain higher contrast-to-noise ratios, little or no toxicity, and rapid clearance for tumor theragnosis in PAI. Currently, the nanoparticles for photoacoustic imaging in NIR-II window can reach the maximum depth of 5 cm under *in vitro* test at the laser energy of 20 mJ/cm^2^ [16]. However, most of their work didn’t validate the *in vivo* optimal imaging depth, especially investigated whole-body biodistribution of nanoparticles in the living body based on PAI [15,16]. And maybe that is the reason why they couldn’t evaluate the toxicity of accumulation through *in vivo* PA imaging at the same time.

The iron-platinum (FePt) alloy nanoparticles (NPs) are superior candidates because of the synergy of both metal elements, platinum and iron. It has excellent superparamagnetic property from iron and X-ray absorption from platinum. Combined with its strong near-infrared (NIR) absorption, it became promising contrast agents in MRI/CT/fluorescence/PA quadruple modal molecular imaging [[17], [18], [19], [20]]. What’s more, with controlled composition, shape, and size, FePt nanoparticles could be modified for diverse biomedical applications [[21], [22], [23], [24], [25]]. Specifically, the surfactants surrounding each FePt nanoparticle can be conjugated with biomolecules like antibodies, proteins, and drugs, rendering the particles better water-soluble, preferable biocompatibility, lower toxicity and more precisely targeted delivery. These features of FePt nanoparticle make it a potential contrasts agent for the targeting PA diagnosis and even magnetothermal [26] or photothermal therapies [27] of breast cancers. But even so, the imaging depth of PAI and the biodistribution of FePt NPs haven’t been carefully evaluated and validated. Here, we conjugated anti-vascular endothelial growth factor receptor (anti-VEGFR) antibody on FePt nanoparticles targeting the neo-vasculatures of breast tumor microenvironments [[28], [29], [30]]. First, verified by fluorescence confocal microscopy and PAI, the anti-VEGFR conjugated FePt NPs can target and detect breast tumors in deep tissues. The depth of *in vivo* PAI is more than 5 cm, which is 2.0 cm deeper than that without contrast agents. In particular, revealed by *in vivo* PAI, the FePt nanoparticles were majorly and quickly metabolized through the liver. Overall, the anti-VEGFR conjugated FePt NPs enable the functional and low-toxicity PAI at few centimeters’ depth, which paves a new path for early detection and even targeting therapy of breast tumor.

## 2 Methods

### 2.1 Materials

Platinum acetylacetonate (Pt(acac)2, ACROS, 97 %), iron pentacarbonyl (Fe(CO)_5_, Aldrich, 99 %), 1,2-hexadecanediol (Aldrich, 90 %), oleyl amine (ACROS, C18 content 80∼90 %), oleic acid (Aldrich, 90 %), 1-octadecene (ACROS, 90 %), cysteamine (Sigma, 95 %), ethyl-3-[3-dimethylaminopropyl] carbodiimide hydrochloride (Aldrich, 98 %), N-hydroxysuccinimide (Acros, 98 %) and anti-VEGFR antibody (Ebioscience, anti-mouse-cd309 biotin, 500 μg/mL)

### 2.2 Synthesis of 12 nm FePt nanoparticles

As-prepared FePt nanoparticles (∼12 nm) were synthesized according to a previous article [20,31]. The typical procedure is described as follows: Pt(acac)_2_ (195 mg), Fe(CO)_5_ (66 μL), 1,2-hexadecandiol (400 mg), oleyl amine (4 mL) and oleic acid (4 mL) and 1-octadecene (4 mL) were loaded into a three-neck flask. The mixture in the N_2_ atmosphere was heated to 240 °C at a heating rate of 15 °C/min. As a result, the mixture was maintained at the refluxing temperature of 240 °C for 45 min before cooling to room temperature. In the further sample collection, the black product was precipitated by adding ethanol, which was used as anti-solvent, and then separated by centrifugation at 3500 rpm. The above separation process was repeated 3∼5 times. Finally, the final product was stored in hexane or toluene for material characterization.

### 2.3 Ligand exchange of 12 nm FePt nanoparticles

In the procedure of ligand exchange, the dry FePt nanoparticles (∼ 50 mg) were dispersed in Dimethyl sulfoxide (10 mL) by sonication. Cysteamine (∼ 1 g) was mixed into the previous mixture at room temperature. The temperature of the mixture was kept at ∼55 °C (heating rate ∼ 5 °C/min) overnight. Finally, the ligand-exchanged nanoparticles were washed to clean the physically adsorbed ligand on the particle surfaces by the addition of methanol and hexane. Then, the ligand-exchanged nanoparticles were collected and stored in a bottle filled with N_2_.

For the anti-VEGFR antibody conjugation of FePt nanoparticles, the anti-VEGFR antibody modified carboxylic group was incubated with ethyl-3-[3-dimethylaminopropyl] carbodiimide hydrochloride (EDC) at 4 °C. After 10 min, a suitable amount of N-hydroxysuccinimide (NHS) was mixed with this solution at 4 °C for 10 min. Then, the cysteamine-modified FePt nanoparticles were added into the above solution and then, the mixture was stirred at 4 °C for overnight. Finally, the pallets were centrifuged in 10,000 rpm for 10 min and washed in phosphate-buffered saline (PBS) twice before the next steps.

### 2.4 *In vitro* and *in vivo* PAI and confocal microscopic imaging

**Fig. 1:**
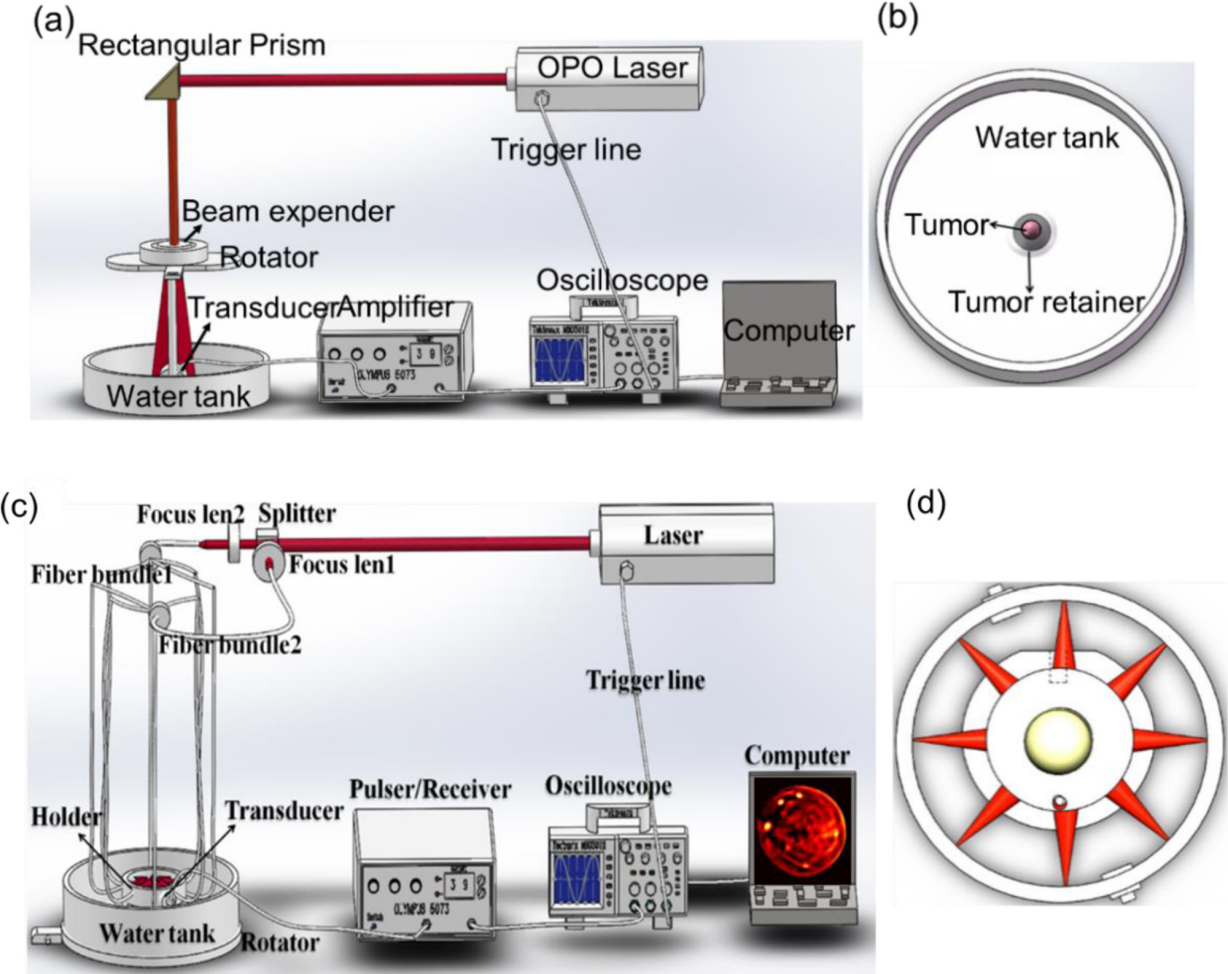
(a) Schematic illustration of the home-made photoacoustic imaging system, and (b) the water tank for mouse tumor experiment; (c) Schematic illustration of the home-made whole-body photoacoustic imaging system, and (d) schematic drawing of the light illumination scheme.

The animal and phantom tests were performed by using the home-made multispectral PAI system and whole-body PAI system (Fig. 1) [2,4]. In this two imaging systems, a pulsed light from an OPO laser (wavelength range from 680 to 1064 nm; pulse duration: 5–10 ns; frequency rate: 20 Hz; Surelite I-10, Continuum) was adopted as a laser source to illuminate the phantom or the animals. To record the PA signals, two 7.5 MHz transducers were circularly rotated by a rotary stage at 360 positions (7.5 MHz central frequency; bandwidth range from 5.03 to 9.46 MHz; V321, Olympus-NDT). The complex wave field signal was first amplified by a Pulser/Receiver (5073R, Olympus) and subsequently converted into digital data. Finally, the images were reconstructed by our developed algorithm. For the phantom experimental tests, the targets with different concentrations of anti-VEGFR conjugated FePt NPs were placed into the solid phantom. For the phantom materials, the agar powder (1–2%) solution was used to solidify the Intralipid as the scatterer. We used India ink as control absorber. Finally, the object-bearing solid phantom was immersed in water to measure the photoacoustic properties of anti-VEGFR conjugated FePt NPs. For all the animal experiments, all the protocols were approved by the Animal Management and Ethics Committee of the University of Macau. The green fluorescence protein (GFP)-labeled 4T1 breast cancer cells were cultured and subcutaneously injected into the back of the nude mice. All the *in vivo* experiments were performed when the tumors reached a size of about 80–140 mm^3^. The GFP-labeled 4T1 breast tumor-bearing mice were intravenously injected with anti-VEGFR conjugated FePt NPs at a dose of 0.8 mg/mL (for 30 g body weight). The 900-nm excited PAI was performed at different time points (0, 0.5, 1, 2, 4, and 6 h) after tail-vein injection. The temperature of the water bath for the PAI system was kept at 37.5 °C. After PAI, the tumors were excised and cryo-sectioned for fluorescence confocal imaging (Carl Zeiss LSM 710 Confocal Scanning Microscope). The GFP contrast of tumor cells and the red fluorescence of FePt NPs were all excited by a 488 nm continuous wave laser.

## 3 Results and discussion

**Fig. 2:**
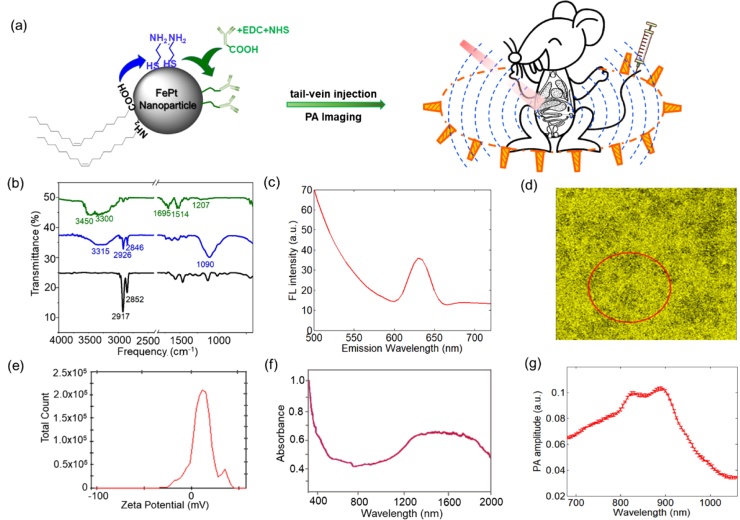
(a) General scheme of the modification of cysteamine and the conjugation of anti-VEGFR antibody on the surface of FePt NP for *in vivo* whole-body PAI. (b) FTIR spectra of as-prepared FePt NPs (black), cysteamine-modified FePt NPs (blue), and anti-VEGFR antibody conjugated FePt NPs (green). (c) The fluorescence spectrum and (d) the corresponding fluorescence image of anti-VEGFR antibody conjugated FePt NPs. (e) The zeta-potential of anti-VEGFR antibody conjugated FePt NPs in water. (f) The absorption [20] and (g) PA spectra of anti-VEGFR antibody conjugated FePt nanoparticles dispersed in water.

**Fig. 3:**
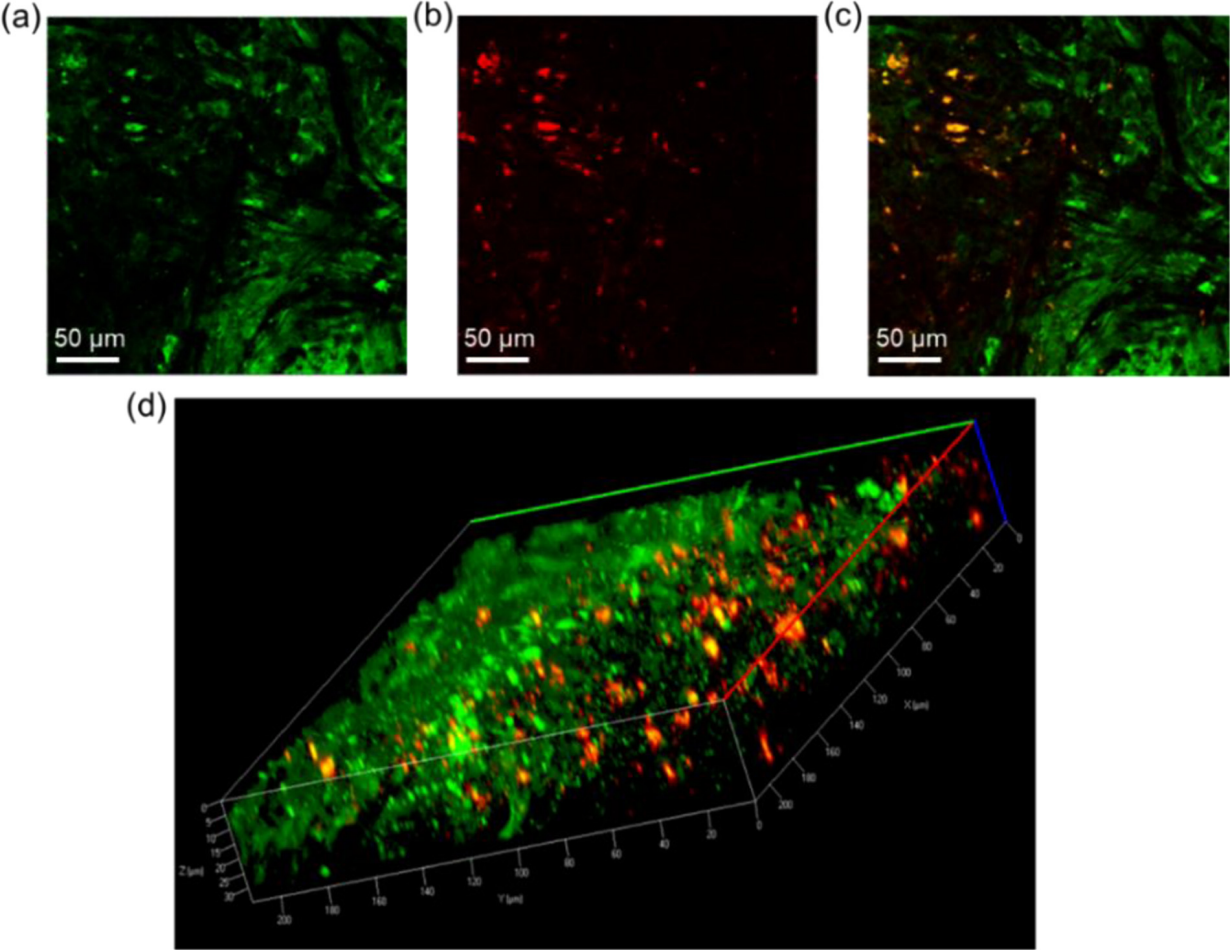
The confocal microscopic imaging of (a) GFP- transfected tumor biopsies, (b) the distribution of anti-VEGFR conjugated FePt NPs, and (c) the merged image in the excised tumor tissue; (d) the 3-D representation of the merged images (220 × 220 × 35 μm).

The as-prepared FePt NPs have sizes of 12.54 ± 1.47 nm (Fig. S1). The HR-TEM images (Fig. S1c) shows the lattice fringes corresponding to a spacing distance of ∼0.19 nm, which are close to the lattice distance of the (200) facet of FePt alloy [32]. The alloying composition of FePt nanoparticles was confirmed by X-ray energy-dispersive spectrometer (Fig. S2a). The results of the alloying composition show that the Pt: Fe ratio is 66:34 for the sample in Fig. S1. Additionally, the typical XRD scan (Fig. S2b) showed the strongest peaks of the (111) and the (200) facet of a face-centered cubic (FCC) structure. Then we performed ligand exchange on the surface of FePt NPs (Fig. 2a). Fourier Transform Infrared (FTIR) spectra of the as-prepared FePt nanoparticles and cysteamine-modified FePt NPs reveal the characteristic bands from the adsorbed ligands, including oleic acid, oleyl amine, and cysteamine, on the surface (Fig. 2b). The FTIR spectrum of as-prepared FePt NPs showed the characteristic bands of symmetric and antisymmetric stretching of CH— vibrational bands at 2852 and 2917 cm^−1^, respectively [31,33]. The spectrum of cysteamine-modified FePt NPs present the significant bands of the C—N stretch and NH— stretching at 1090 cm^-1^ and 3315 cm^−1^, respectively [17,20]. Further, the conjugation of anti-VEGFR antibody onto the FePt NPs was achieved by the reaction of the amine group of cysteamine on the nanoparticle surface with carboxylic group of the antibody (Fig. 2a). The ligand exchanges of FePt nanoparticles with cysteamine and anti-VEGFR antibody were confirmed from the characteristic bands of 1207 (C—N stretch), 1514 (CN—), 1695 (NH_2_ scissoring), 3300 and 3450 (N—H_2_ groups of antibody) cm^−1^ [34,35]. Furthermore, the average surface zeta potential of anti-VEGFR conjugated FePt NPs is approximately 10.6 mV (Fig. 2e), indicating that the anti-VEGFR conjugated FePt NPs are stable in aqueous solutions. These data, together with good water solubility, ensure the complete and successful ligand exchange process. Then we tail-vein injected the particles to see whether they can target the breast-tumors in the animal model. Under 488 nm excitation, the anti-VEGFR conjugated FePt NPs showed a red fluorescence peak around 635 nm (Fig. 2c). In the 488 nm excited fluorescence confocal image of cryo-sectioned tumor tissues, this contrast of FePt NPs can reveal the targeted distribution of particles (red color in Fig. 3) in the microenvironment of GFP-labeled tumor cells (green color in Fig. 3). These results demonstrated that the anti-VEGFR conjugated FePt nanoparticles have good targeting ability for breast tumors. For the PA imaging, we examined the photoacoustic spectrum of anti-VEGFR conjugated FePt NPs (Fig. 2g). It showed an optimal response at 900 nm excitation, where FePt NPs have large absorption (Fig. 2g & f). In Fig. 2f and g, the deviations between the PA and absorption spectra profiles have been widely observed for photoacoustic imaging contrast agents, which should mainly be caused by two factors: (1 optical absorption and PA spectra measure different photophysical processes; and (2 optical illumination parameters are different, high-power pulsed laser for PA spectra *versus* low-power continuous-wave light illumination for absorption spectra [36,37].

It demonstrates that the nanoparticles have high-efficiency photothermal conversion and can serve as effective PAI contrast agent.

**Fig. 4:**
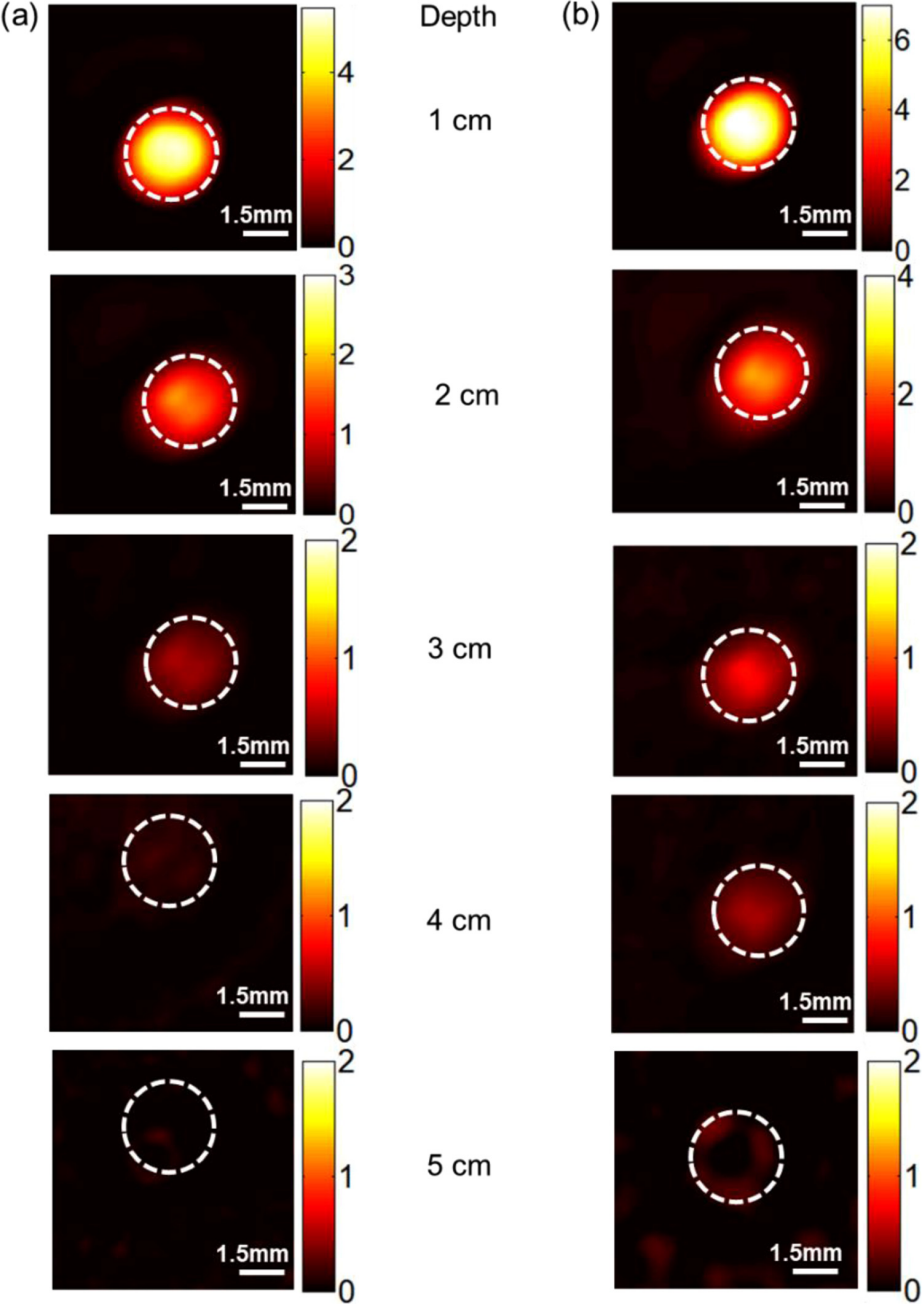
The *in vitro* photoacoustic imaging results of absorber without Fept nanoparticles (a) and with Fept nanoparticles (b) at different depths at the laser energy density of 20 mJ/cm^2^.

To evaluate the PAI depth of FePt NPs, we first conducted the *in vitro* phantom experiment. The control group filled the light absorber in a 3 mm-diameter cylinder containing 1% Intralipid, India ink, distill water, and Agar powder. By controlling the dosage of India ink, the absorption coefficient of the light absorber was tuned to 0.4 mm^−1^ at the wavelength of 900 nm, which is close to that of breast cancer tissues. The experimental group prepare same light-absorbing cylinder and added 100 μL FePt nanoparticles (0.8 mg/mL) into it. The laser energy density for PAI is 20 mJ/cm^2^, which meets the safety criterion (≤20 mJ/cm^2^) of the American National Standards Institute (ANSI). During the experiment, we put different thickness of chicken breast tissues on the light-absorbing phantom to test the imaging depth. We found that the PA signals of the light-absorbing target decreased with the increase of buried depth (Fig. 4). The imaging depth of the FePt NPs reached 5 cm (Fig. 4b), which is 2.0 cm deeper than that of the control sample (Fig. 4a).

**Fig. 5:**
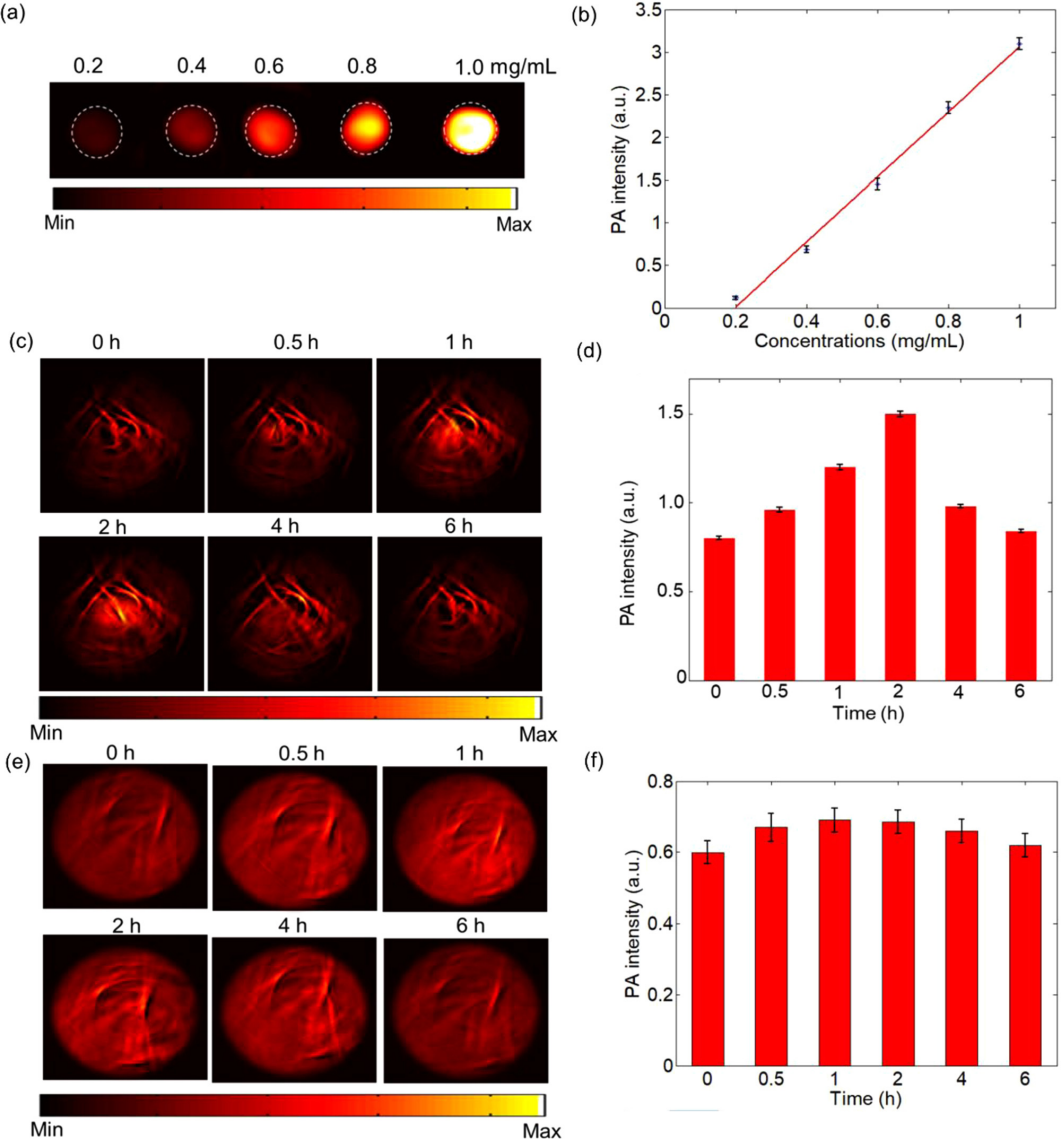
The photoacoustic imaging results. (a) *In vitro* PA imaging results and (b) Linear relationship between PA signal and concentration of the anti-VEGFR conjugated FePt nanoparticles. (c) *In vivo* PA imaging of tumor tissue before and after tail injection of the anti-VEGFR conjugated FePt nanoparticles under 900 nm laser irradiation at different time points (0, 0.5, 1, 2, 4 and 6 h post-injection). (d) Normalized PA signals in tumors at different time points. (e) *In vivo* PA imaging of region without tumor before and after tail injection of the anti-VEGFR conjugated FePt nanoparticles under 900 nm laser irradiation at different time points (0, 0.5, 1, 2, 4 and 6 h post-injection). (f) Normalized PA signals in region without tumor at different time points.

Then we examined the PA properties of the anti-VEGFR conjugated FePt NPs in the home-made multispectral PAI system (Fig. 1a). At 900 nm excitation, the sensitivity of PAI detection was around 0.2 mg/mL (Fig. 5a and b). After the intravenous injection of anti-VEGFR conjugated FePt NPs, there is a significant rise of PA signal in the tumor region from 0 to 2 h post-injection (Fig. 5c and d). Compared with region without tumor, there is only a slight rise of PA signal in the region from 0 to 6 h after injection (Fig. 5e and f). It indicates a targeted delivery of NPs by enhanced retention and permeation and the affinity binding of anti-VEGFR ligands. The PA signals started to decay after 2 h and returned to the background level at 6 h post-injection (Fig. 5d), suggesting an easy and rapid clearance of targeting-enriched FePt NPs in the tumor microenvironment.

**Fig. 6:**
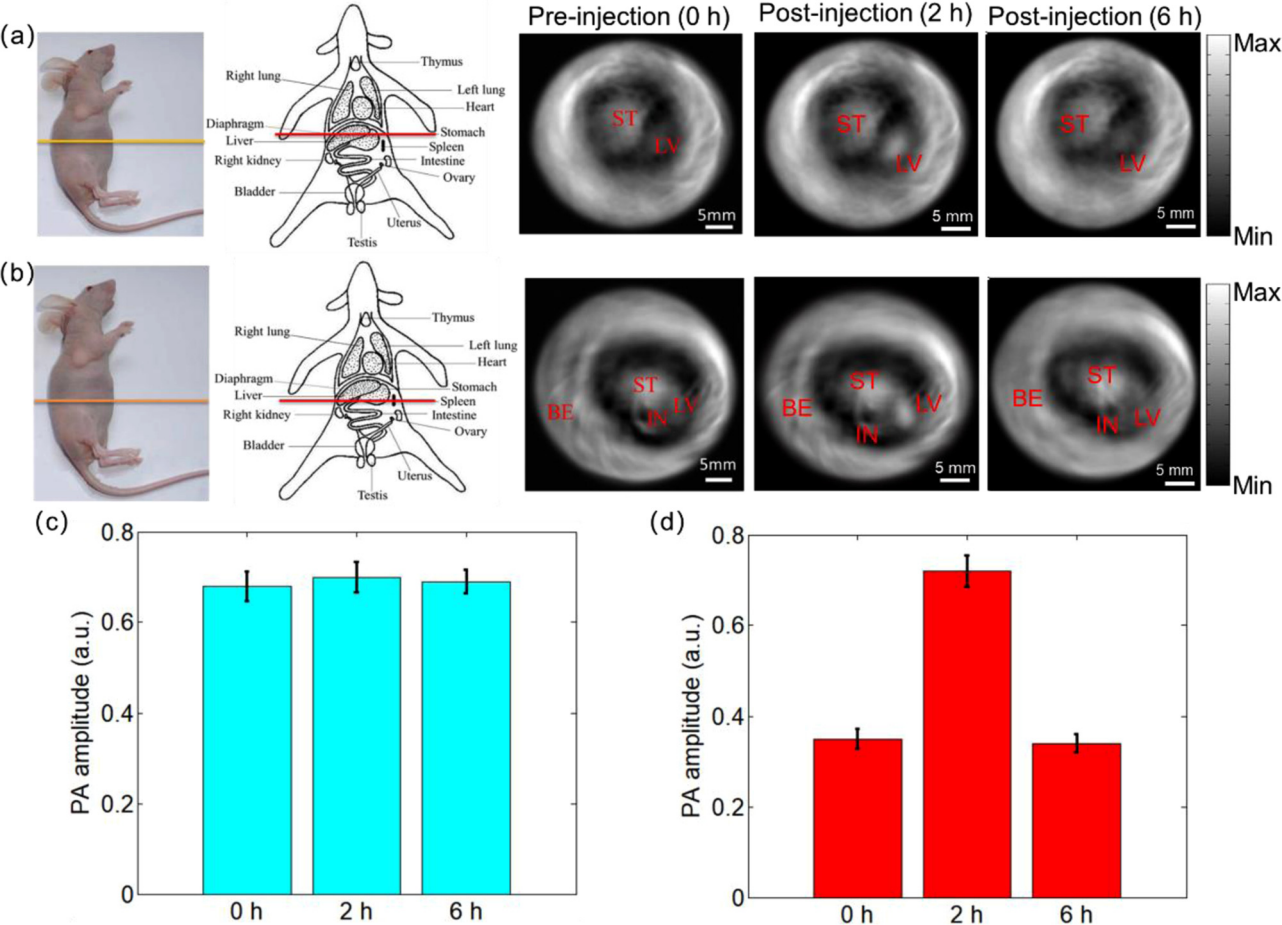
The whole-body photoacoustic imaging of mouse. (a) and (b): The PA imaging results of different cross-sections and different time points in mice, respectively. The analysis of the PA intensity at (c) stomach (light blue bars) and (d) liver (red bars) at the time point of pre-injection of nanoparticles, 2 h post-injection, and 6 h post-injection. ST: Stomach; LV: Liver; BE: Blood Vessels; IN: Intestine.

Finally, for the evaluation of nanopartilces metabolism, we investigated the biodistribution and clearance dynamics of FePt NPs in mice with *in vivo* PAI. Due to deep imaging depth of PAI, our system can make whole-body tomography imaging at different cross-section of mice (Fig. 6a and b). Comparing the PA signals before and after injection, we found the PA signals in stomach (ST) didn’t change a lot, while the liver (LV) showed an obvious accumulation at 2 h post-injection. This indicates that the injected FePt NPs can be effectively cleared through liver within 6 h after injection. This PAI-based biodistribution analysis also demonstrates that we can achieve 3-cm deep PAI of FePt NPs in soft tissues.

## 4 Conclusion

In brief, we successfully developed low-toxicity FePtNPs for few centimeters deep photoacoustic imaging of breast tumors. We first validated the targeting of anti-VEGFR conjugated FePt NPs by the multiphoton microscopy and *in vivo* PA imaging. We also verified that the anti-VEGFR conjugated FePt NPs could serve as photoacoustic imaging contrast agents for the *in vivo* detection of breast cancer. At low laser energy density of 20 mJ/cm^2^ (≤ ANSI safety limit), the *in vitro* imaging depth could extend over 5 cm with the help of FePt NPs. After a tail-vein injection, their dynamic biodistribution and tumor targeting were *in vivo* visualized by a whole-body PA imaging. The FePt NPs accumulated in the tumor region from 0 to 2 h after injection and then were rapidly cleared from the tumor sites after 6 h. We found the cleared nanoparticles were majorly and fleetly metabolized through the liver. These results validated the low-toxicity and clinical potential of the FePt NPs in the PA theragnosis of breast cancer.

## Declaration of Competing Interest

This manuscript has not been published or presented elsewhere in part or in entirety and is not under consideration by another journal. All the contributing authors have reviewed this paper. The study design only involved animal studies and was approved by the appropriate ethics review board. We have read and understood your journal’s policies, and we believe that neither the manuscript nor the study violates any of these. There are no conflicts of interest to declare.
