# Supplementary material for: Low-toxicity FePt nanoparticles for the targeted and enhanced diagnosis of breast tumors using few centimeters deep whole-body photoacoustic imaging
Source: Photoacoustics. 2020 Apr 11;19:100179. doi: 10.1016/j.pacs.2020.100179 (PMC7168769; doi:10.1016/j.pacs.2020.100179)
Supplement: Supplementary file 1 [file mmc1.docx]

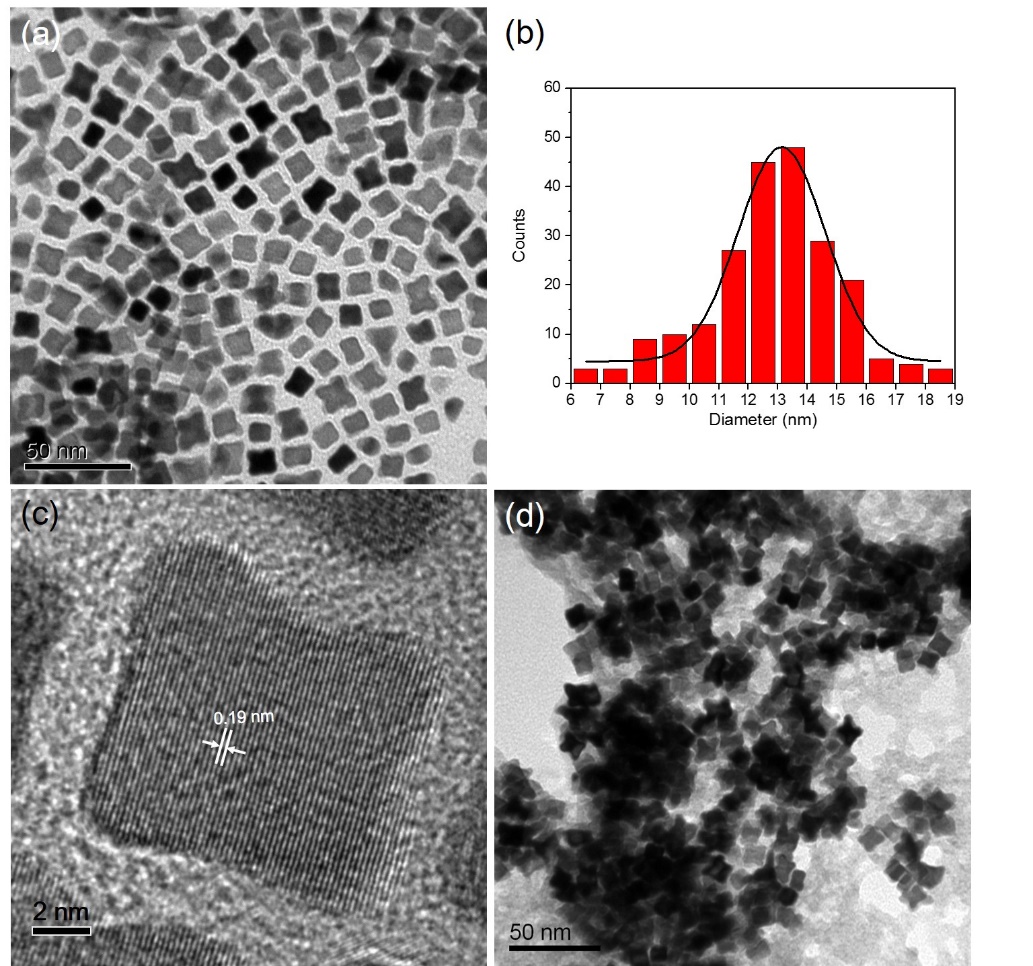


**Figure S1.** (a) TEM images obtained from the as-prepared FePt nanoparticles. (b) The size distribution acquired from 200 as-prepared FePt nanoparticles. (c) HR-TEM images obtained from the as-prepared FePt nanoparticles. (d) TEM images obtained from the cysteamine-modified FePt nanoparticles.


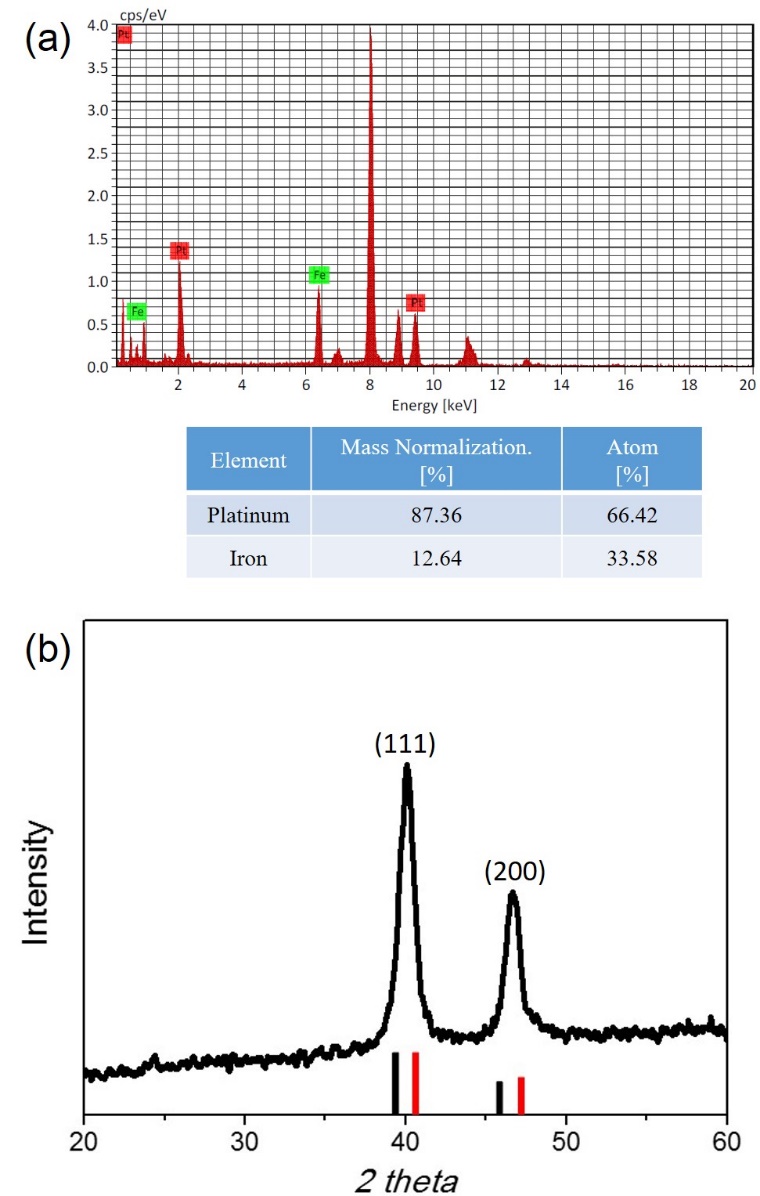


**Figure S2.** (a) X-ray energy-dispersive spectrum of FePt nanoparticles (b) The powder x-ray diffraction scan of FePt nanoparticles. Note: For comparison, the patterns of Pt (black line) and FePt (red line) as reference.
